# An online human–robot collaborative grinding state recognition approach based on contact dynamics and LSTM

**DOI:** 10.3389/fnbot.2022.971205

**Published:** 2022-09-02

**Authors:** Shouyan Chen, Xinqi Sun, Zhijia Zhao, Meng Xiao, Tao Zou

**Affiliations:** ^1^School of Mechanical and Electrical Engineering, Guangzhou University, Guangzhou, China; ^2^Department of Rehabilitation Medicine, Zhujiang Hospital, Southern Medical University, Guangzhou, China

**Keywords:** contact dynamics, online classification, collaborative grinding, physical human–robot collaboration, human intent classification

## Abstract

Collaborative state recognition is a critical issue for physical human–robot collaboration (PHRC). This paper proposes a contact dynamics-based state recognition method to identify the human–robot collaborative grinding state. The main idea of the proposed approach is to distinguish between the human–robot contact and the robot–environment contact. To achieve this, dynamic models of both these contacts are first established to identify the difference in dynamics between the human–robot contact and the robot–environment contact. Considering the reaction speed required for human–robot collaborative state recognition, feature selections based on Spearman's correlation and random forest recursive feature elimination are conducted to reduce data redundancy and computational burden. Long short-term memory (LSTM) is then used to construct a collaborative state classifier. Experimental results illustrate that the proposed method can achieve a recognition accuracy of 97% in a period of 5 ms and 99% in a period of 40 ms.

## Introduction

Human–robot collaboration has attracted the attention of researchers (Golz et al., 2015). Human–robot contact recognition is one of the critical issues for physical human–robot collaboration (PHRC) (Cherubini et al., 2016; Labrecque et al., 2017). Previous studies have mainly focused on the detection of a collision between humans and a robot (Billard and Kragic, 2019). Many types of observers have been proposed, including torque observer, energy observer, momentum observer, state observer based on electromyography (EMG) or touch, etc. (Haddadin et al., 2017; Losey et al., 2018). Different types of contact occur during human–robot collaboration (e.g., intentional interaction and accidental interaction), and early collision detection is not able to identify the different types of contact (Althoff et al., 2012). Intentional human interaction expects an active robot collaboration, while accidental collision calls for an immediate halt of the machine (Kang et al., 2019). In view of this, scholars attempt to identify the intention of physical human–robot interaction (PHRI) (Olivares-Alarcos et al., 2019). Many studies focused on identifying the occurrence, position, and direction of the interaction, without discussing the difference in dynamics between the human–robot interaction and the robot–environment contact. Without identifying the difference in dynamics between the human–robot contact and the robot–environment contact, it is difficult to precisely identify whether a robot is in contact with the environment or a human, especially when a robot is in contact with both the environment and a human in a similar direction.

This paper proposes a dynamics-based approach to identify the PHRC state. The main idea of the proposed approach is to analyze the dynamics of PHRC and find out an appropriate dynamic feature to classify the human–robot collaborative state. For this purpose, robotic grinding is selected as an example of PHRC and the corresponding dynamic model is established. The inputs of a classifier are generated in accordance with the dynamic model. A human–robot collaborative grinding state classifier is constructed based on long short-term memory (LSTM). To reduce data redundancy and increase reaction speed, feature selections based on Spearman's correlation and random forest recursive feature elimination are performed. The main contributions of this project are:

- Dynamic characteristics of the PHRC are used to generate features for PHRC grinding state recognition.
- Feature selections based on Spearman's correlation and random forest recursive feature elimination are performed to reduce data redundancy and increase reaction speed. LSTM is used to design a collaborative state classifier.

This paper is organized as follows. Section Related works presents related research. Section Human–robot collaborative grinding model presents models of PHRC grinding. Section Online collaborative state classifier presents an online human–robot collaborative grinding state classifier. Section Experimental validation illustrates experimental results. Section Conclusion presents conclusions to this paper.

## Related works

Many approaches have been proposed to identify human intentions during human–robot collaboration, including vision-based classifiers, EMG-based classifiers, contact force-based classifiers, and multimodal fusion-based classifiers (Ajoudani et al., 2018). Some scholars attempted to design a state observer to detect the collision between a human and a robot (Zhou and Wachs, 2018; Zhang et al., 2021). These methods focused on identifying the occurrence and position of the human–robot contact. Cherubini et al. (2016) elucidated that the alternation of active and passive behaviors occurs frequently during human–robot collaboration, which requires a robot to actively collaborate with humans. The premise of active robot collaboration is the accurate and quick realization of human intentions (Villani et al., 2018; Veselic et al., 2021). In view of this, many scholars began to study the intention of a human–robot interaction (Veselic et al., 2021). Lanini et al. (2018) used the arm position and interaction force data during human–human collaboration to develop a multiclass classifier, enabling a robot to recognize human intentions during the collaborative task of carrying an object. Golz et al. (2015) acquired a set of human–robot contact features by analyzing the physical contact model and the real impact process. Using these data, an SVM-based classification system was constructed to identify intended and unintended human–robot contact. However, their contact model ignores damping, stiffness, and kinetic energy. Therefore, their classifier is only suitable for static or low-speed robotic tasks. Considering the difficulties in obtaining an accurate human–robot interaction model, Li and Ge (2014) used a neural network to construct an online intention estimation approach to estimate human motion intentions. Relying on human force and pose measurements, Cremer et al. (2020) proposed a neural network-based estimation method to predict human motion intentions. Yan et al. (2019) proposed an intentional classification model based on the radial basis function neural network, which can deduce human intentions, according to the dynamic behaviors of humans and robots within previous collaboration tasks.

Apart from the human–robot interaction, the robot–environment contact may occur during PHRC. It is difficult to recognize the human–robot contact when a robot is performing a contacting task. To address this, Lippi and Marino (2020) constructed a filter based on the robot–environment contact model to filter out the robot–environment contact force and to detect additional forces, e.g., human–robot contact force. The advantage of their method is that even if the robot is in contact with both the environment and humans in a similar direction, the human–robot contact can be recognized. However, this approach requires that the robot–environment contact force should be known in advance and the force fluctuation should be limited. Designing a human–robot collaboration dynamics-based classifier may be a viable way to obtain a universal human–robot collaborative state classifier.

Human collaborators always change their limb impedance or stiffness during human–robot collaboration. Upon this knowledge, Yu et al. (2020) used the least squares method to learn the impedance of the human body. Their study only focused on the adjustment of robot impedance control parameters to achieve collaboration compliance without discussing the value of human body dynamics for PHRI state recognition. Geravand et al. (2013) considered that an unexpected collision between a human and a machine can be defined as a hard contact between rigid bodies, while the human–robot contact can be defined as a soft contact. According to their analysis, a hard contact results in a high-frequency signal and a soft contact results in a low frequency signal. Based on this, Geravand designed a high-low pass filter to identify human–robot contact intention. Furthermore, Losey et al. (2018) clarified that human intentions during the human–robot contact are continuous and time-varying, which result in a non-linear contact force.

**Figure 1:**
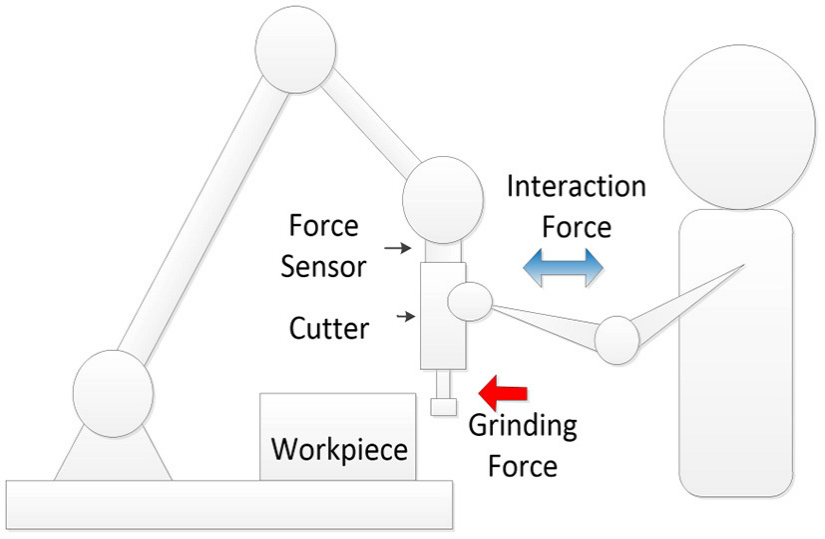
Human–robot collaborative grinding.

Up to now, only a few studies have focused on distinguishing between the human–robot and the robot–environment contact. The main issue is the impact of the fluctuation of contact force between the robot and the environment on the recognition of the human–robot contact. To address this problem, this paper proposed a dynamics-based approach for distinguishing the human–robot contact and the robot–environment contact. Experimental set-up is illustrated in Figure 1. The human–robot contact and the robot–environment contact occur at the same time in the similar direction.

## Human–robot collaborative grinding model

### Robot grinding dynamic model

Robot grinding dynamic model can be expressed as,


(1) $$\begin{array}{l} MẌ+CẊ+KX=G\left(X,u\right)+F_{c}+F_{r} \end{array}$$

where *F*_*c*_ is the grinding force; *M*, *C*, and *K* are the system mass, damping and stiffness matrices; are the grinding depth and its first and second derivatives; *u* is motor voltage; is the contact force caused by motor voltage and grinding depth; *F*_*r*_ is external disturbance force; and *F*_*r*_is robotic grinding force, which can be expressed as (Chen et al., 2019).


(2) $$\begin{array}{l} F_{c}=F_{d}+F_{g} \end{array}$$

where *F*_*d*_ is the deformation force caused by compression between a grinding tool and a workpiece; *F*_*g*_ is cutting force, which is affected by the grinding depth, feed rate, and rotation speed of a grinding tool. As frequent contacts between a grinding tool and a workpiece occur during robotic grinding, it is not efficient to construct a robot–environment contact model based on the additional contact constraint method. Therefore, the continuous contact force model based on the Hertz contact theory is used in this paper, which can be expressed as (Zhang et al., 2020).


(3) $$\begin{array}{l} F_{g}=K_{e}\sigma^{\alpha }\left(\sigma \geq 1\right) \end{array}$$

where σ is normal embedding depth; α is exponential coefficient of deformation; and *K*_*e*_ is equivalent stiffness. Assuming that the main deformation is on a grinding tool. The grinding tool deformation has a relationship with the removal rate and feed rate, which can be expressed as,


(4) $$\begin{array}{l} \sigma =\int \left(Ẋ\left(t\right)-{Ẋ}_{r}\right)dt \end{array}$$

where Ẋ is the feed rate and Ẋ_*r*_ is the removal rate. The cutting force produced by the revolution of a grinding tool can be regarded as a periodic force:


(5) $$\begin{array}{l} F_{d}\left(t\right)\text{=}F_{d}\left(t+T_{w}\right) \end{array}$$

where $T_{w}\text{=}\frac{T}{b}$ is the cutting period of a blade, $T_{w}\text{=}\frac{1}{v_{w}}$ is the rotation period of a grinding tool, and *v*_*w*_ is the rotation speed of a grinding tool. According to the traditional grinding force formula, grinding force is influenced by cutting depth, feed speed, tool rotation speed, and grinding area, which can be expressed as,


(6) $$\begin{array}{l} F_{d}\left(t\right)=F_{p}X^{\alpha }{Ẋ}^{\beta }{V_{w}}^{\gamma }S^{\delta }=F_{d}\left(X,Ẋ,V_{w},S\right). \end{array}$$

Combining Equations (1)–(6), the robotic grinding model can be expressed as,


(7) $$\begin{array}{l} MẌ+CẊ+KX=G\left(X,u\right)+K_{e}\sigma^{\alpha }+F_{d}\left(X,Ẋ,T_{w},S\right)+F_{r} \end{array}$$

### A dynamic model of human–robot collaborative grinding

Human collaborators always adjust their muscles and change the stiffness or impedance of their body to control contact force, achieving a compliance collaboration. The human–robot collaborative grinding model can be expressed as,


(8) $$\begin{array}{lll} MẌ+CẊ+KX & = & G\left(X,u\right)+K_{e}\sigma^{\alpha }+F_{d}\left(X,Ẋ,T_{w},S\right)+F_{r}+F_{h} \end{array}$$

where *F*_*h*_ is an external force exerted by humans. Considering the human arm as a spring-damping system and *F*_*h*_ can be calculated by,


(9) $$\begin{array}{l} F_{h}=-D_{h}\left(t\right)ẋ\left(t\right)+K_{h}\left(t\right)\left(x_{d}\left(t\right)-x\left(t\right)\right) \end{array}$$

where *x*_*d*_ is the desired location expected by a human collaborator, *D*_*h*_ and *K*_*h*_ are the damper and stiffness matrices of the human arm and may vary or fluctuate during human–robot collaboration. Assuming that *D*_*h*_ and *K*_*h*_ non-linearly and continuously fluctuate within a bounded range, the fluctuation of *F*_*h*_ is also non-linear, continuous, and bounded. Therefore, Δ*F*_*h*_, ΔḞ_*h*_, and $\Delta {\bar{F}}_{h}$ are used to identify the human–robot interaction state.


(10) $$\begin{array}{l} \Delta F_{h}=-\Delta D_{h}\left(t\right)ẋ\left(t\right)+\Delta K_{h}\left(t\right)\left(x_{d}\left(t\right)-x\left(t\right)\right) \end{array}$$

(11) $$\begin{array}{l} \Delta {\bar{F}}_{h}\text{=}{\left(\text{Mean}\Delta F_{h}\right)}_{\max}-{\left(\text{Mean}\Delta F_{h}\right)}_{\min} \end{array}$$

where represents the force slope caused by Δ*D*_*h*_Δ*K*_*h*_, which is non-linear and continuous and $\Delta {\bar{F}}_{h}$ represents the mean value range of slope fluctuation.

### Problem description

**Figure 2:**
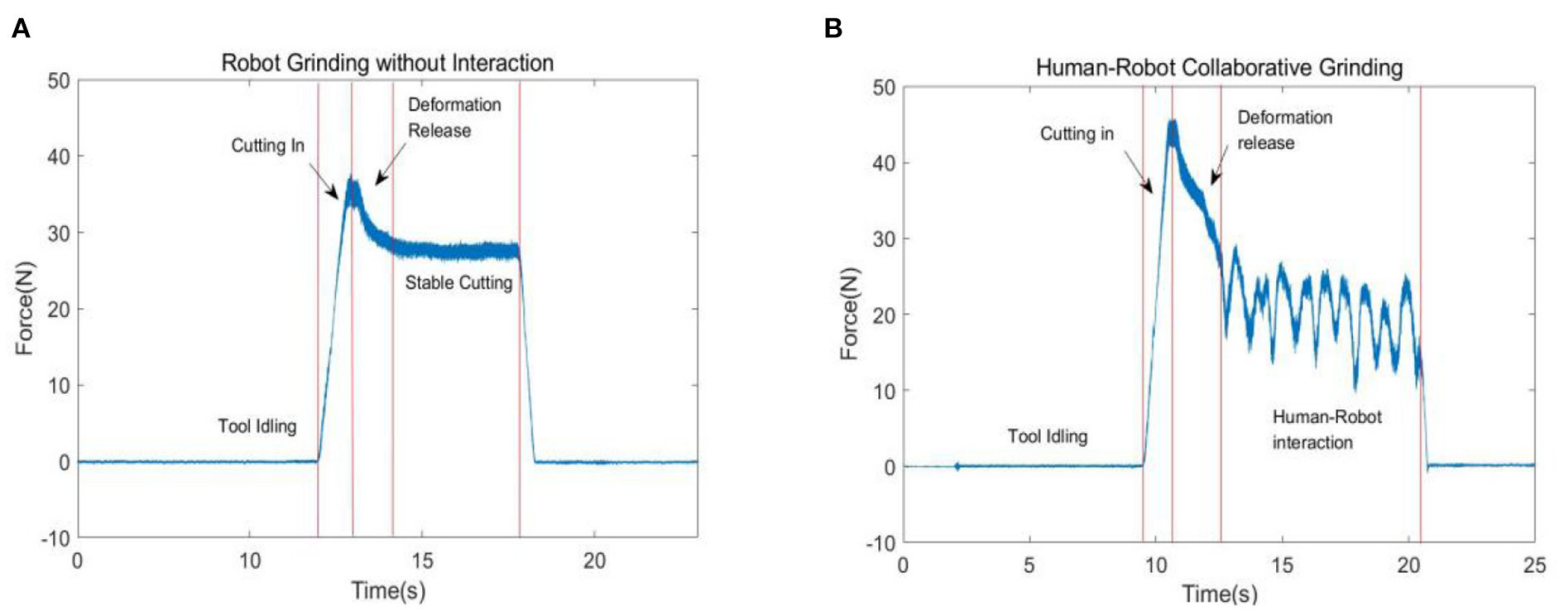
Collaborative state labeling. **(A)** Robotic grinding states and **(B)** human–robot collaborative grinding states.

According to Equation (10) and the human–robot collaborative grinding process, five states can be defined, namely, tool idle state, cutting-in state, deformation release state, stable cutting state, and human–robot collaborative cutting state. These states are shown in Figures 2A,B.

To precisely identify the human–robot collaborative state, it is essential to analyze the characteristics of each state. The first step is to clearly describe the signal characteristics of each collaborative grinding state. According to the discussion above, the definition of a feature of each state is presented in Table 1.

**Table 1:** Feature of each state.

**State**	**State feature**
Tool idling	No contact force; a periodic force fluctuation is caused by cutter eccentricity
Cutting-in	Extrusion deformation and force between workpiece and cutter increase dramatically. Since contact is between rigid workpiece and flexible grinding tool with constant stiffness, the force curve is linear.
Deformation release	Cutting begins and the deformation is still large. Extrusion force decreases as material of workpiece being removed
Stable cutting	Contact force between workpiece and cutter is stable, as feed rate is equal to removal rate.
Human–robot collaborative grinding	Human–robot interaction force occur, contact force changes non-linearly

According to the features of each state as presented in Table 1, the characteristics can be concluded.

After concluding the characteristics of each state, the next step is to construct a framework for collaborative state classification.

## Online collaborative state classifier

Compared with offline classification, an online classifier is required to achieve quick and accurate classification with the least data and computation. More precisely, a collaborative robot requires a reaction at the millisecond level. Therefore, the issues need to be considered are classification accuracy, reacting speed, and data amount.

### Overall process and feature acquisition

In this section, an overall structure of the human–robot collaborative grinding state classification is introduced. To develop this scheme, two kinds of grinding data are collected by the force sensor installed between the robot end effector and a grinding tool, including robotic grinding data and human–robotic collaborative grinding data.

#### Classification scheme

**Figure 3:**
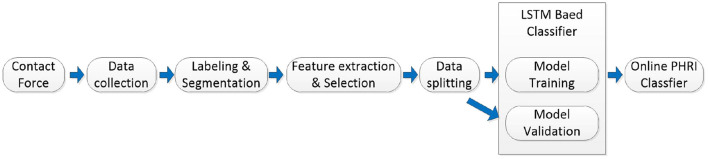
Schematic diagram of classification process.

An overall structure of the proposed online human–robot collaborative grinding scheme is shown in Figure 3. Robotic grinding force and human–robot contacting force data are first collected, labeled, and divided into segments. The force data segments are then used to extract the features of different collaborative states. Feature selections are conducted after feature extraction to reduce data redundancy and increase reaction speed. The selected features are then put into an LSTM-based classifier to train the classifier model. Model parameters are later used to construct an online classifier of the PHRC grinding state.

#### Data collection

**Figure 4:**
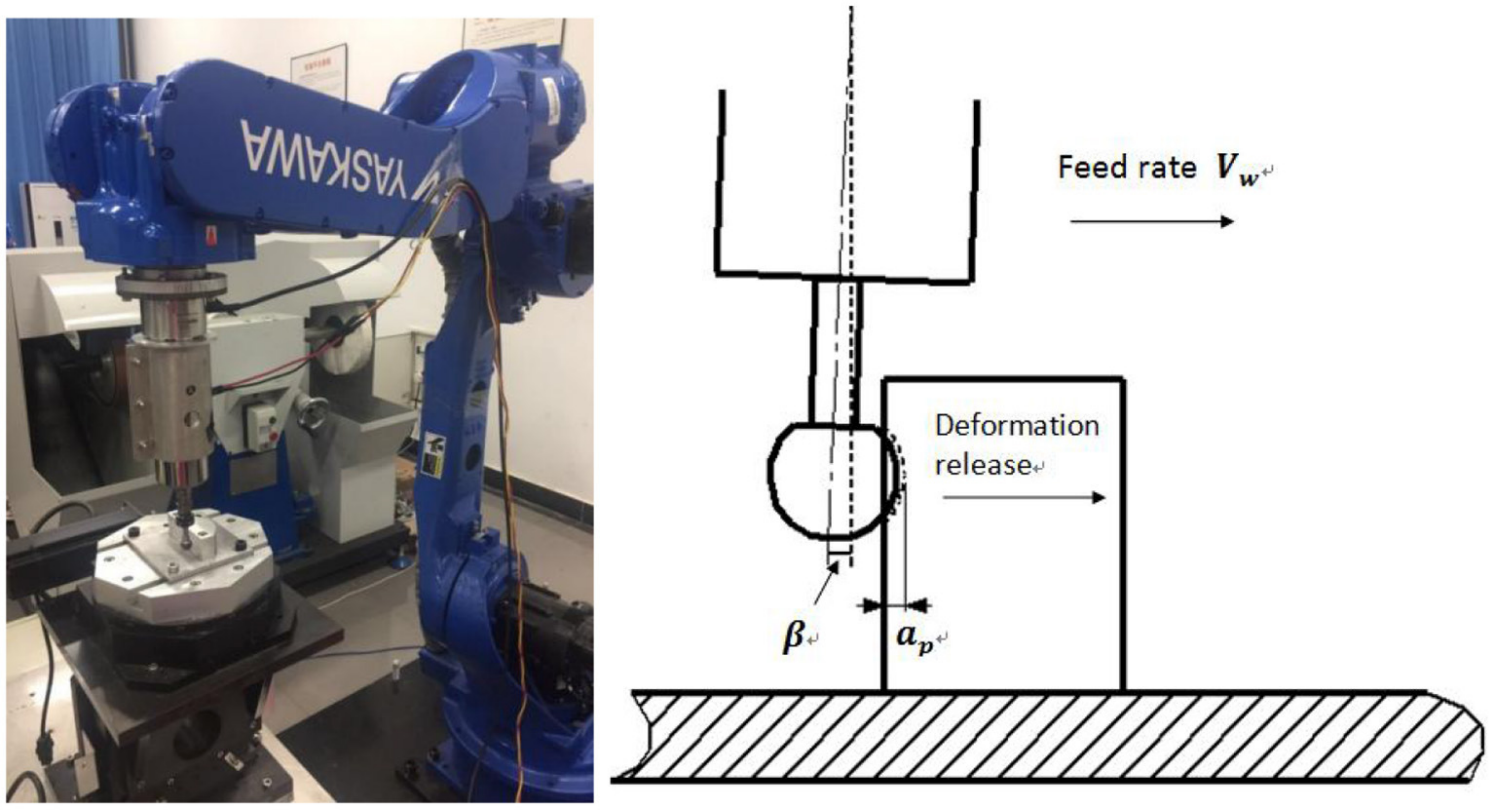
Robotic grinding system.

To construct a human–robot collaborative grinding data set, a six-degrees-of-freedom Yaskawa robot grinding system, as shown in Figure 4, is constructed. A six-degree force sensor is mounted between a grinding tool and the robot end effector to collect contact force data during human–robot collaborative grinding. The rotation speed of a grinding tool is 2,000 rpm. During robotic grinding, an experimenter exerts intentional contact on the clamping position of a grinding tool. Different PHRC states are conducted to obtain the data set.

##### Signal segmentation and sample labeling

After collecting human–robot collaborative grinding force signals, segmentation is performed to split force signals into segments with a fixed length *l*. The segment should be long enough to contain sufficient information to classify states and short enough to achieve a fast reaction. Therefore, the demand for reaction accuracy and speed influences the determination of *l*. The classification of experiments with different values of *l* is to explore the impact of *l* value on classification accuracy and speed. According to grinding tool rotation speed and the characteristics presented in Table 2, 11 values of *l* are set, which are 5, 10, 20, 30, 40, 50, 60, 70, 80, 90, and 100 ms. In the segmentation process, invalid data caused due to the damage in the storage or an unrecognizable PHRI signal are eliminated. To avoid the deviation in results caused by uneven data distribution, the data were homogenized. Finally, 14,300 data are obtained and used for collaborative state classification experiments.

**Table 2:** Characteristics of each state.

**State**	**Force mean amplitude**	**Force fluctuation**	**Equivalent stiffness**
Tool idling	0	Little	No change
Cutting-in	Dramatical and linear increase	Large	No change
Deformation release	Considerable and non-linear decrease	Large	Regular change
Stable cutting	Little fluctuation	Little	No change
Human–robot collaborative grinding	Large fluctuation	Large	Irregular change

#### Feature extraction

Time-series signal features can be classified into time domain and frequency domain features. Time domain features include mean, variance, average amplitude, energy source, root mean square, root square amplitude, standard deviation (SD), peak coefficient, shape coefficient, skewness, pulse factor, margin factor, kurtosis, etc. Peak coefficient, shape coefficient, skewness, impulse factor, margin factor, and kurtosis are dimensionless time domain characteristics. Common frequency domain features include barycentric frequency, mean square frequency, root mean square frequency, frequency variance, frequency SD, etc. Considering the computing time required by frequency domain feature generation, only 12 time domain features are preselected for human–robot collaborative state classification, which are mean value, variance, average amplitude, energy source, root mean square, root amplitude, SD, shape parameter, skewness, pulse factor, margin factor, and kurtosis. Apart from these features, the contact force slope, slope fluctuation range, and force slope change speed are also considered.

##### Feature selection

To reduce feature redundancy and select the critical features for state classification, Spearman's correlation and recursive feature elimination based on random forest (RF-RFE) are used to evaluate the preselected features.

Spearman's correlation analysis can identify the dependency between any two features. It is not only suitable for linear correlation cases but also suitable for non-linear cases. Suppose there are two sums of eigenvalues:


$$\begin{array}{l} X=\left\{X_{1},X_{2},...,X_{m}\right\}\\ Y=\left\{Y_{1},Y_{2},...,Y_{m}\right\} \end{array}$$

where *m* is the data set size. Spearman's correlation coefficient can be calculated by,


$$\begin{array}{l} \rho_{X,Y}=\frac{\sum_{i=1}^{m}\left(x_{i}-\bar{x}\right)\left(y_{i}-ȳ\right)}{{\left(\sum_{i=1}^{m}{\left(x_{i}-\bar{x}\right)}^{2}\sum_{i=1}^{m}{\left(y_{i}-ȳ\right)}^{2}\right)}^{1/2}} \end{array}$$

where $\bar{.}$ represents the mean value. ρ_*X,Y*_ is the coefficient describing the dependency of *X* on *Y*.

##### Random forest-based recursive feature elimination

**Figure 5:**
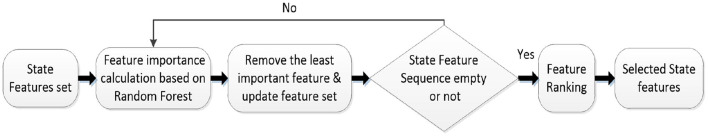
Recursive feature elimination based on random forest (RF-RFE) algorithm flow.

Recursive feature elimination based on random forest is used to calculate the importance of features and selected features. The RF-RFE algorithm flow is shown in Figure 5 (Shang et al., 2021). Firstly, random forest model is established, and feature samples are randomly extracted based on the Bootstrap sampling technique. The impact of feature samples on classification accuracy is then evaluated to identify the importance of the selected feature to state classification. The least important features are removed, and the evaluation is reconducted by random forest with the remaining features. After that, the abovementioned steps are reconducted until the ranking of state features is obtained.

### Deep learning-based classifier

#### LSTM-based classifier

**Figure 6:**
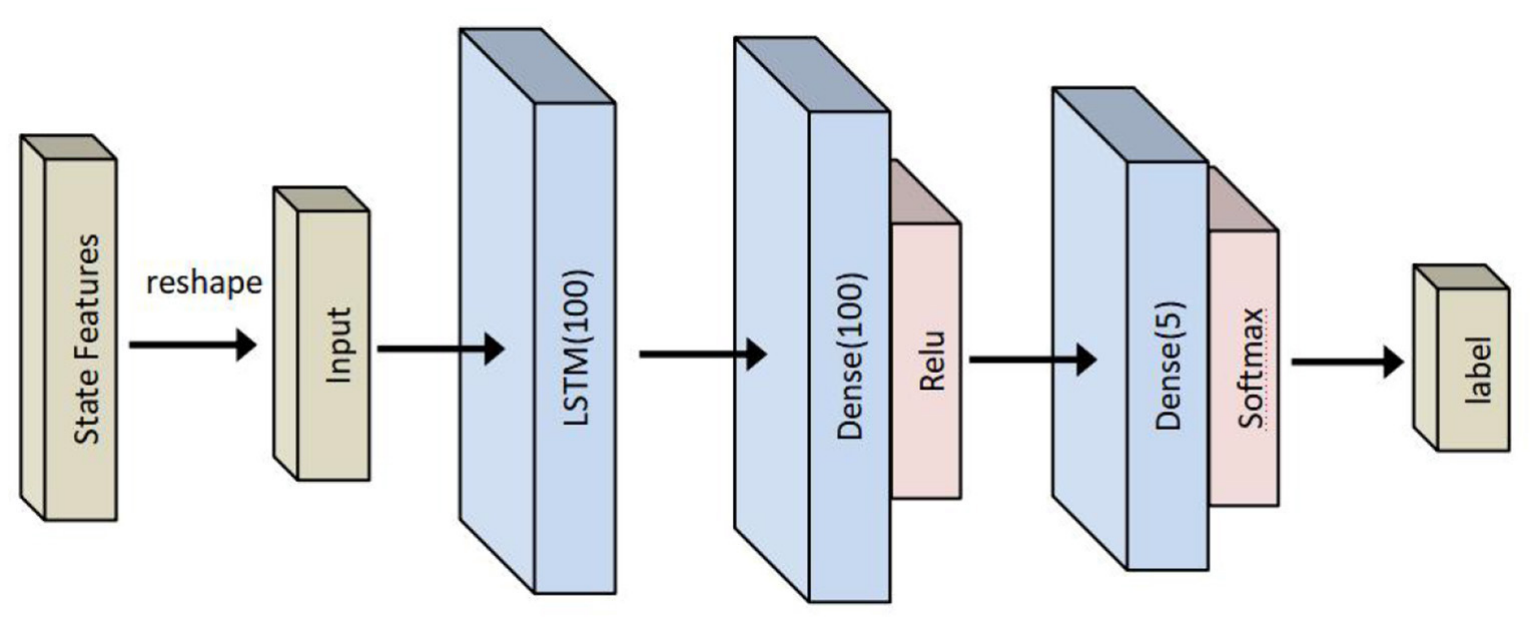
Long short-term memory- (LSTM-) based human–robot collaborative grinding state classifier.

The structure of an LSTM classifier used in this paper is shown in Figure 6 and is composed of one LSTM layer and two sense layers. The LSTM classifier is used to study the relationship between inputs and collaborative states. Therefore, the inputs of an LSTM classifier are the selected features mentioned above, and the output is a label of collaborative states.

#### LSTM fully convolutional network based classifier

**Figure 7:**
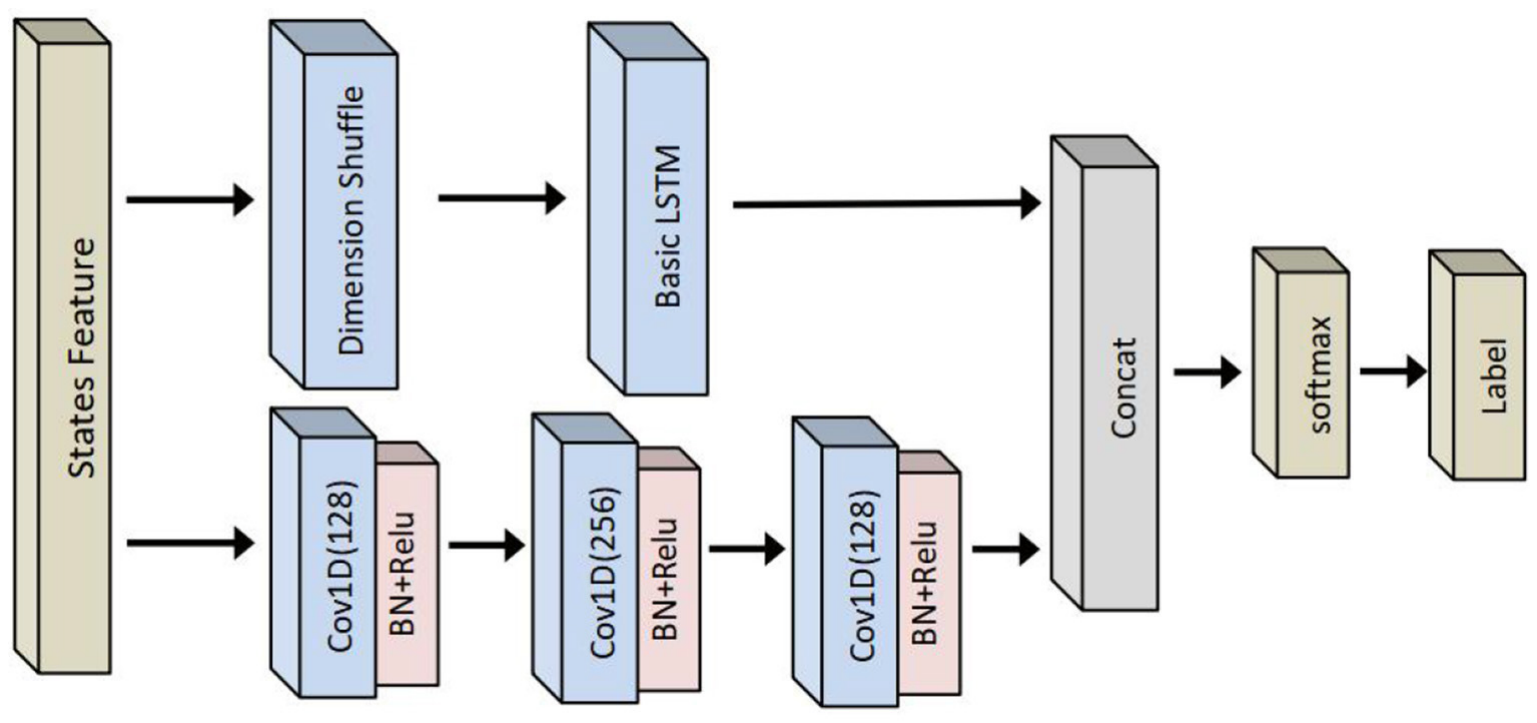
LSTM fully convolutional network (FCN-) based human–robot collaborative grinding state classifier.

For a comparison, long short-term memory FCN-based classifier is also evaluated. The performance of LSTM-FCN has been verified on public time series data sets (Karim et al., 2018). Its structure is shown in Figure 7, which is formed by paralleling LSTM and time convolution models. The selected features are first input to LSTM and CNN models. The LSTM model consists of a dimension transformation layer, an attention layer, a single-layer LSTM, and a dropout layer, while the CNN model consists of three time convolution networks with a filter size of 128, 256, and 128, respectively. Each convolution layer is normalized and followed by the Relu activation function. Finally, global pooling is carried out after the final convolution block.

## Experimental validation

To verify the validity of the proposed approach, experiments of collaborative state classification are conducted. The first step is to extract 15 kinds of features. All the samples are randomly shuffled and split into a training set and a test set with the partition ratio 3:1. For classifier training, cross entropy is selected as loss function when the number of iterations is 2,000. The learning rate is set to 1e-2, and the reduction speed is 1e-4. To facilitate discussion, three groups of features are defined:

- Group 1 has domain features only, including contact force mean, variance, average amplitude, energy source, root mean square, root square amplitude, SD, peak coefficient, shape coefficient, skewness, pulse factor, margin factor, and kurtosis.
- Group 2 consists of the features in Group 1 and contact dynamic features Δ*F*_*h*_, ΔḞ_*h*_, and $\Delta \;{\bar{F}}_{h}$.
- Group 3 consists of the features selected from Group 2.

### Collaborative state classification based on time domain features

Time domain features included in Group 1 are used as inputs to classify the human–robot collaborative grinding state without considering the human–robot contact dynamics. The impact of segment length *l* and feature numbers on the performance of collaborative state recognition is explored. For a convenient discussion, time domain features are labeled with numbers 1–13 corresponding to contact force mean, variance, average amplitude, energy source, root mean square, root square amplitude, SD, peak coefficient, shape coefficient, skewness, pulse factor, margin factor, and kurtosis in the time domain features. Experimental results are presented in Table 3. The vertical axis of a table is segment length, while the horizontal axis is the time domain feature number.

**Table 3:** Long short-term memory- (LSTM-) based physical human–robot collaboration (PHRC) state recognition (Group 1 features; %).

**Length (ms)/feature**	**1-2**	**1-3**	**1-4**	**1-5**	**1-6**	**1-7**	**1-8**	**1-9**	**1-10**	**1-11**	**1-12**	**1-13**
5	84.7	84.9	84.4	84.0	83.6	86.2	88.3	90.0	90.1	90.8	90.7	90.9
10	89.2	89.2	90.1	90.7	91.0	91.2	91.1	92.5	93.0	93.1	93.4	93.8
20	90.9	91.2	91.1	92.1	92.4	93.1	93.0	94.5	94.4	94.2	94.2	94.5
30	92.7	93.1	92.9	93.2	93.6	94.7	94.2	95.5	96.1	96.9	96.4	96.7
40	93.3	93.5	93.9	94.2	94.7	95.4	95.7	96.6	97.0	97.4	97.7	97.9
50	94.9	95.2	95.1	95.4	96.0	96.8	96.2	97.3	98.0	98.6	98.4	98.4
60	95.0	95.6	95.7	95.6	96.2	96.8	97.5	97.7	98.3	98.4	99.1	99.4
70	95.1	95.6	96.1	96.0	96.7	97.1	97.7	98.1	98.7	99.0	99.3	99.3
80	96.3	96.5	97.1	97.6	98.1	98.5	98.1	97.9	99.0	99.1	99.2	99.5
90	96.5	97.1	97.4	97.5	98.0	98.3	98.4	98.9	99.0	99.4	98.9	99.0
100	96.3	96.9	97.8	97.8	97.6	98.6	98.7	98.9	99.2	99.1	99.4	99.7

It can be observed from Table 3 that, while the segment length is 5 ms, the recognition accuracy of the collaborative state increases roughly as the number of features increases, from 84.7% at 1–2 to 90.9% at 1–13. It should be noticed that obvious improvements in accuracy occur at 1–7 and 1–9. Starting with 1–9, although the number of features increases, the accuracy does not change significantly, which means that features 9–13 may have a little impact on recognition accuracy. In other words, some features can be eliminated to reduce feature redundancy and computing burden. In general, recognition accuracy increases as the segment length and the feature number increase, and the earliest 99% occur at 60 ms and features 1–12.

### Collaborative state classification based on time domain features and contact dynamics

Features contained in Group 2 are then used as classifier inputs for collaborative state recognition. It can be seen that the highest recognition accuracy at the 5-ms stage is 96.4%, which is 5% higher than the one presented in Table 4. The highest recognition accuracy is 99.6% appearing at 100 ms and features 1–13, which is almost the same as the one presented in Table 4. However, the earliest 99% appear at 40 ms and 1–12 features, which is 20 ms earlier than the one presented in Table 4. The contact dynamics features can increase the recognition accuracy of classifier in the early stage. In other words, contact dynamics-based classifier can achieve a quick and precise classification of the human–robot collaborative grinding state with little computational burden.

**Table 4:** LSTM-based PHRC state recognition (Group 2 features; %).

**Length (ms)/feature**	**1-2**	**1-3**	**1-4**	**1-5**	**1-6**	**1-7**	**1-8**	**1-9**	**1-10**	**1-11**	**1-12**	**1-13**
5	95.4	95.5	95.6	95.5	95.7	95.6	96.0	96.1	96.4	96.0	96.2	96.1
10	96.0	95.7	95.7	96.0	95.7	95.7	96.1	96.9	96.8	96.5	97.0	96.8
20	96.0	95.8	96.5	96.2	96.2	96.5	96.1	96.2	96.3	96.4	97.3	97.3
30	95.9	96.5	97.0	97.2	97.4	97.5	97.4	97.2	97.6	97.3	98.8	98.1
40	97.2	97.6	97.8	97.6	97.6	97.9	97.5	98.2	98.1	97.7	99.0	98.7
50	98.0	98.4	98.2	98.4	98.2	98.4	98.3	98.4	98.6	98.7	99.1	99.1
60	98.2	98.1	98.4	98.1	97.9	98.1	97.9	98.4	98.2	98.5	98.7	99.2
70	98.2	98.1	98.1	98.5	98.4	98.4	98.1	98.8	98.9	98.2	98.3	99.4
80	98.1	98.4	98.4	98.3	98.2	98.3	98.4	98.5	98.5	98.6	98.5	99.3
90	98.3	98.4	98.4	98.4	98.4	98.4	98.3	98.2	98.3	98.3	99.5	99.6
100	98.4	98.4	98.4	98.4	98.5	98.4	98.1	98.0	98.5	98.6	99.6	99.6

### Collaborative state classification based on feature selection

**Figure 8:**
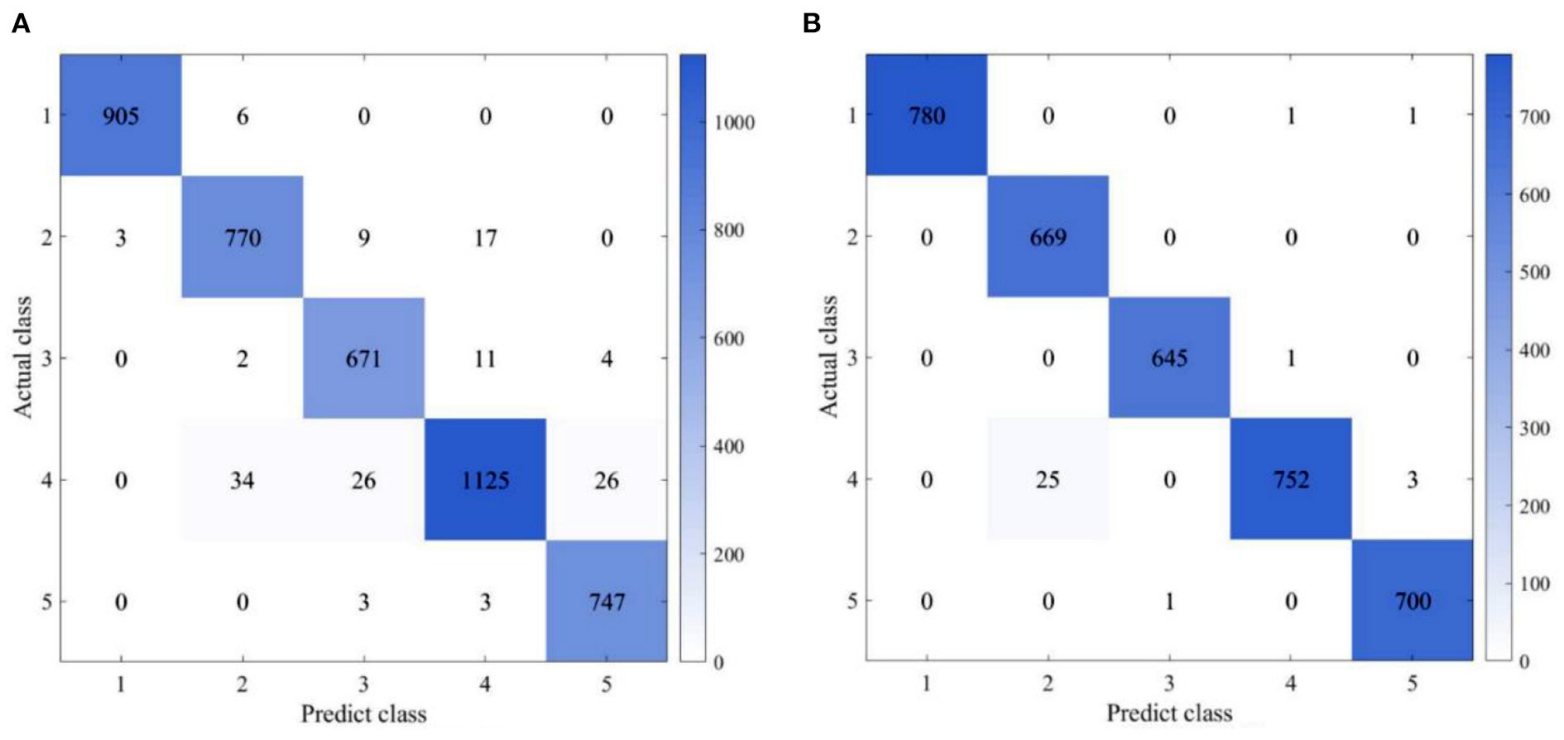
Confusion matrix (LSTM-based classification; **(A)** 5 ms and **(B)** 100 ms).

To further reduce computational burden, feature selections are then conducted based on the Spearman's correlation analysis and recursive feature elimination method. According to the Spearman's correlation analysis, the correlation coefficient between average amplitude and root square amplitude, root mean square, energy source, and peak coefficient is more than 0.9. Therefore, the root square amplitude, root mean square, energy source, and peak coefficient are represented by the average amplitude. The recursive feature elimination method based on random forest is also conducted. The remaining features include mean, variance, average amplitude, SD, and human–robot contact dynamics. Therefore, the selected features are mean value, variance, average amplitude, SD, and human–robot contact dynamic features Δ*F*_*h*_, ΔḞ_*h*_, and $\Delta {\bar{F}}_{h}$. As presented in Table 5, although some features are removed, an LSTM-based state classifier can achieve a recognition accuracy of 97.0% in 5 ms and 99.4% in 100 ms. Compared with the ones presented in Table 5, there is no considerable decrease in recognition accuracy, which illustrates that feature selections can reduce computational burden while ensuring recognition accuracy. The confusion matrix, as shown in Figure 8, indicates that misclassifications occur in the distinction of cutting-in and human–robot contact states.

**Table 5:** LSTM and LSTM-FCN-based PHRC state recognition (Group 3 features; %).

**Length (ms)**	**5**	**10**	**20**	**30**	**40**	**50**	**60**	**70**	**80**	**90**	**100**
LSTM	97.0	97.7	98.0	98.4	98.5	99.2	98.8	98.9	99.0	99.0	99.4
LSTM-FCN	94.7	96.4	96.2	96.6	96.4	96.7	96.7	98.3	98.7	98.7	99.1
LSTM (without feature extraction)	82.5	83.5	83.6	83.3	83.4	84.6	83.7	84.7	83.7	84.0	85.1

LSTM-FCN-based state recognition is also carried out to evaluate the performance of LSTM-based recognition. Compared with an LSTM-based classifier, the recognition accuracy obtained by an LSTM-FCN-based classifier is 2% lower on average from 5 to 60 ms. However, the structure of an LSTM-based classifier is simpler than one of the LSTM-FCN-based classifiers, which can be observed from Figures 6, 7. In other words, the number of parameters is less in an LSTM-based classifier than in LSTM-FCN-based classifiers. Therefore, while performing online recognition, an LSTM-based classifier can achieve lower computing time and cost compared with an LSTM-FCN-based classifier. Finally, LSTM-based state recognition experiments without feature extraction are conducted. As presented in Table 5, the highest recognition accuracy of the LSTM-based state without feature extraction is only 85%, which is 14% lower than one of the proposed approaches.

## Conclusion

In this paper, a contact dynamics-based collaborative state recognition approach for human–robot collaborative grinding is proposed. Human–robot and robot–environment contact dynamics are analyzed. The result is used to extract human–robot collaborative grinding state features. Considering the computational burden and reaction speed required for online collaborative state recognition, feature selections based on the Spearman's correlation analysis and the recursive feature elimination method are performed. LSTM is finally used to construct a collaborative state classifier. Experimental results illustrate that the proposed approach can achieve a classification accuracy of 96.7% in 5 ms and 99.4% in 100 ms. The limitation of this paper is that the environment and a workpiece are assumed to be rigid. While a workpiece is flexible or soft, contact dynamics will be different and difficult to describe.

## Data availability statement

The original contributions presented in the study are included in the article/supplementary material, further inquiries can be directed to the corresponding authors.

## Author contributions

All authors listed have made a substantial, direct, and intellectual contribution to the work and approved it for publication.

## Funding

This work was supported in part by the National Natural Science Foundation of China under Grants 51905115, 12172095, and 51775122.

## Conflict of interest

The authors declare that the research was conducted in the absence of any commercial or financial relationships that could be construed as a potential conflict of interest.

## Publisher's note

All claims expressed in this article are solely those of the authors and do not necessarily represent those of their affiliated organizations, or those of the publisher, the editors and the reviewers. Any product that may be evaluated in this article, or claim that may be made by its manufacturer, is not guaranteed or endorsed by the publisher.
